# Plasma kynurenine to tryptophan ratio and arginine as biomarkers associated with GOLD classification and predicting poor prognosis in patients with acute exacerbation of chronic obstructive pulmonary disease

**DOI:** 10.3389/fmed.2026.1807281

**Published:** 2026-05-07

**Authors:** Yongjian Tian, Xue Li, Li He, Shunshun Cui, Pengjuan Liu, Wei Wang

**Affiliations:** 1 Department of Laboratory Medicine, Fuyang People's Hospital, Fuyang, Anhui, China; 2 Department of Respiratory and Critical Care Medicine, Fuyang People's Hospital, Fuyang, Anhui, China

**Keywords:** acute exacerbation of chronic obstructive pulmonary disease, arginine, GOLD classification, kynurenine, kynurenine to tryptophan ratio, prognosis, tryptophan

## Abstract

**Background:**

Acute exacerbation of chronic obstructive pulmonary disease (AECOPD) is life-threatening with limited prognostic biomarkers. The kynurenine pathway and arginine (Arg) metabolism are linked to COPD inflammation, but their relevance to GOLD classification and prognosis in AECOPD remains elusive. This study aimed to explore plasma kynurenine (Kyn) to tryptophan (Trp) ratio (KTR) and Arg as potential prognostic biomarkers.

**Methods:**

176 AECOPD patients (Fuyang People’s Hospital, May 2022–March 2024) and 60 age/sex-matched healthy controls were enrolled. AECOPD patients were grouped into poor (80 cases: 1-year death/readmission) and good (96 cases) prognosis groups, all receiving 2022 GOLD-guided treatment. Plasma Kyn, Trp, and Arg were quantified via UPLC-MS/MS. Correlations of KTR/Arg with GOLD classification, their prognostic value, and plasma metabolic differences among the three groups were analyzed.

**Results:**

Compared with the good prognosis group, the poor prognosis group exhibited significantly higher Kyn and KTR levels, a higher proportion of GOLD grade 3/4 patients, and lower Trp and Arg levels (all *p* < 0.05). Violin plot analysis further revealed that both AECOPD subgroups had elevated Kyn/KTR and reduced Trp/Arg relative to healthy controls, with more severe metabolic dysregulation in the poor prognosis cohort (all *p* < 0.05). Spearman correlation analysis indicated a positive correlation between GOLD grade and KTR (*r* = 0.747, *p* < 0.05) and a negative correlation with Arg (*r* = −0.638, *p* < 0.05), and KTR was inversely correlated with Arg in both prognostic subgroups (good: *r* = −0.597; poor: *r* = −0.620, both *p* < 0.001). Multivariate Logistic regression identified Kyn (OR = 1.759), KTR (OR = 2.862), Trp (OR = 0.449), and Arg (OR = 0.843) as independent predictors of poor prognosis in AECOPD patients. ROC curve analysis demonstrated that KTR (AUC = 0.724) and Arg (AUC = 0.774) had good predictive performance, and their combined detection achieved a higher AUC of 0.826.

**Conclusion:**

Plasma KTR and Arg levels in AECOPD patients are positively correlated with severe pulmonary function and poor prognosis, indicating a close association with prognosis and supporting their potential as novel biomarkers for prognostic stratification in this population.

## 1 Introduction

Chronic obstructive pulmonary disease (COPD) is a chronic airway inflammatory disease characterized by progressive decline in pulmonary function and persistent respiratory airflow limitation (1). Acute exacerbation of COPD (AECOPD), defined as an acute worsening of respiratory symptoms beyond the normal day-to-day variation, is associated with severe pulmonary infections, rapid deterioration of pulmonary function, and increased risk of respiratory failure, leading to poor clinical outcomes (2, 3). Frequent AECOPD-related hospitalizations have been linked to elevated mortality rates, highlighting the urgent need for reliable biomarkers to assess the severity of pulmonary function impairment and predict prognosis in AECOPD patients.

Kynurenine (Kyn), an intermediate metabolite of the essential amino acid tryptophan (Trp), plays a critical role in regulating the proliferation and differentiation of reactive T lymphocytes, as well as modulating immune suppression and amplifying inflammatory responses (4). Previous studies have demonstrated that inflammatory states accelerate Trp metabolism, resulting in increased Kyn levels and a higher Kynurenine to Tryptophan Ratio (KTR), a robust biomarker reflecting the inflammatory and immune status of the host (5). Arginine (Arg), a basic amino acid, is also involved in attenuating inflammatory responses and regulating immune function (6, 7). However, the association between plasma KTR/Arg levels, the severity of pulmonary function impairment, and clinical outcomes in AECOPD patients remains poorly understood.

This study aims to explore the associations between the plasma levels of KTR and Arg at admission and the Global Initiative for Chronic Obstructive Lung Disease (GOLD) classification and 1-year prognosis in patients with AECOPD from the perspective of metabolomics. It provides a theoretical basis for predicting the decline in pulmonary function in AECOPD patients and for individualized treatment to reduce mortality and the readmission rate of patients.

## 2 Materials and methods

### 2.1 General information

This study enrolled AECOPD patients treated at Fuyang People’s Hospital between May 2022 and March 2024. All patients received standardized guideline-based treatment for acute exacerbations per the 2022 GOLD guidelines, with core therapeutic principles aligned with the 2024 version, including oxygen therapy to maintain peripheral oxygen saturation at 90–92%, short-acting bronchodilators as first-line therapy (nebulized salbutamol 5 mg every 6–8 h for monotherapy, with ipratropium bromide 0.5 mg added for combination therapy in case of suboptimal response), antibiotics prescribed based on sputum culture and susceptibility results or Chinese AECOPD management guidelines, and equivalent-dose systemic corticosteroids (40 mg intravenous methylprednisolone once daily for 5–7 days, or 30–40 mg oral prednisone daily for mild exacerbations). Additional supportive treatments were administered as clinically indicated. This study adhered to the Declaration of Helsinki and was approved by the Medical Ethics Committee of Fuyang People’s Hospital.

**Figure 1:**
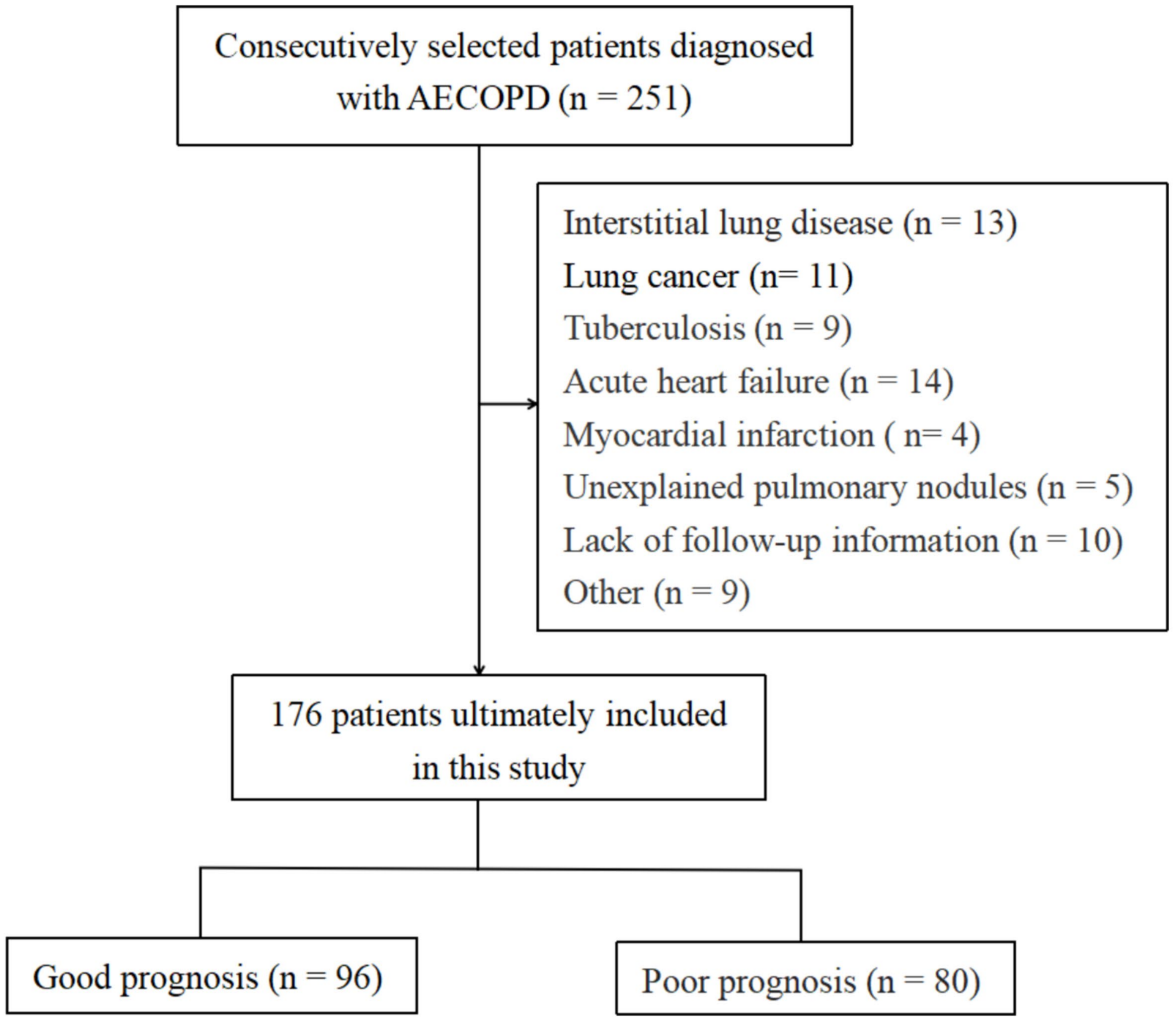
Flowchart of participants’ inclusion and exclusion criteria.

*Inclusion criteria*: (1) The included patients met the diagnostic criteria for AECOPD (8) and absence of a current or historical diagnosis of asthma requiring regular controller therapy or documented evidence of bronchial hyperresponsiveness in adulthood; (2) The clinical medical records were detailed and complete; (3) The patients had not used immunosuppressive drugs before admission. *Exclusion criteria*: (1) Patients with COPD in the stable phase; (2) Patients with other respiratory diseases such as pulmonary fibrosis, pulmonary malignant tumors, pulmonary inflammatory nodules, or a confirmed diagnosis of asthma-COPD overlap (ACO) per the Global Initiative for Asthma (GINA) and GOLD joint definition; (3) Patients with heart diseases such as end-stage diseases like acute heart failure and myocardial infarction; (4) Patients who had used supplementary amino acid drugs within 3 months and Patients with mental disorders and communication disorders. The patient screening process is summarized in Figure 1. Detailed general and medical history information of each subject was collected through medical record review or telephone follow-up, including gender, age, course of disease, smoking history, drinking history, past medical history, and detailed pulmonary function test data. See Table 1 for details.

**Table 1:** Comparison of general clinical data and research indicators between the two groups.

Variable	Good prognosis group (*n* = 96)	Poor prognosis group (*n* = 80)	*p* value
Age (year)	71.74 ± 9.24	73.97 ± 8.06	0.092
Sex/*n* (%)			0.069
Male	61 (63.54%)	61 (76.25%)	
Female	35 (36.46%)	19 (23.75%)	
Duration of disease (year)	5.00 (1.00, 10.00)	9.00 (3.00, 14.00)	0.052
Hypertension/*n* (%)			0.180
Yes	28 (29.17%)	31 (38.75%)	
No	68 (70.83%)	49 (61.25%)	
Coronary atherosclerotic heart disease/*n* (%)			0.558
Yes	24 (25.00%)	17 (21.25%)	
No	72 (75.00%)	63 (78.75%)	
Diabetes mellitus/*n* (%)			0.085
Yes	5 (5.21%)	10 (12.50%)	
No	91 (94.79%)	70 (87.50%)	
Cerebral infarction/*n* (%)			0.186
Yes	27 (28.13%)	30 (37.50%)	
No	69 (71.87%)	50 (62.50%)	
Drink alcohol/*n* (%)			0.250
Yes	29 (30.21%)	18 (22.50%)	
No	67 (69.79%)	62 (77.50%)	
Smoking index (pack-years)	200.00 (45.00, 4500.00)	350.00 (75.00, 600.00)	0.065
Hospitalization time (day)	8.04 ± 2.63	8.09 ± 2.91	0.913
Time to readmission (months)	3.3 ± 1.4	-	-
GOLD classification/*n* (%)			<0.001
Grade 1/2	57 (59.38%)	17 (21.25%)	
Grade 3/4	39 (40.62%)	63 (78.75%)	

GOLD, Global Initiative for Chronic Obstructive Lung Disease; Time to readmission (months)—only for poor prognosis group.

### 2.2 Sample collection and detection method

After admission, 3 mL of fasting venous blood was collected from each patient using EDTA anticoagulant tubes for plasma separation. The blood was centrifuged at 3500 rpm for 10 min to separate plasma, which was then stored in an ultra-low temperature freezer at −80 °C to avoid repeated freezing and thawing of the samples.

Analytical Instrument: plasma concentrations of Kyn, Trp, and Arg were quantified using an ultra-performance liquid chromatography–tandem mass spectrometry (UPLC-MS/MS) system (Waters Corporation, Milford, MA, USA). Mass Spectrometry Parameters: Ionization mode: Electrospray ionization (ESI) positive ion mode. Acquisition mode: Multiple reaction monitoring (MRM). Chromatographic Conditions: Column: Cortecs UPLC C18 column (1.7 μm particle size, 2.1 mm × 150.0 mm; Waters Corporation). Mobile phase: Phase A: 0.1% (v/v) formic acid in ultrapure water; Phase B: 0.1% (v/v) formic acid in acetonitrile (HPLC grade). Flow rate: 500 μL/min; Column temperature: 60 °C; Injection volume: 2 μL; Data Processing: Metabolomics data were processed using MassLynx V4.2 software (Waters Corporation). Reagents and Standards: All analytical reagents and standards were obtained from Sigma-Aldrich (St. Louis, MO, USA) and ANPEL Laboratory Technologies (Shanghai, China), and handled strictly following the manufacturers’ protocols.

### 2.3 Pulmonary function testing

Timing and Instrumentation: Pulmonary function tests were performed on all enrolled patients during their clinical stable phase using a Master Screen Diffusion spirometer (Jaeger, Würzburg, Germany). The primary outcome was forced expiratory volume in 1 s as a percentage of predicted value (FEV₁%pred), and all procedures were strictly conducted according to the American Thoracic Society/European Respiratory Society (ATS/ERS) standard protocols (9). Severity Grading: Patients were stratified into four grades based on the GOLD severity classification criteria (10): Grade 1: FEV₁%pred ≥ 80%. Grade 2:50% ≤ FEV₁%pred < 80%. Grade 3: 30% ≤ FEV₁%pred < 50%. Grade 4: FEV₁%pred < 30%.

### 2.4 Clinical outcomes

The primary endpoint was defined as the clinical outcome at the end of the 1-year follow-up period. Poor prognosis was identified as readmission due to AECOPD or death from treatment failure, whereas good prognosis was defined as the absence of either event.

### 2.5 Statistical analysis

Data analysis was performed using SPSS 27.0 statistical software. The sample size was calculated using PASS 11 software. Count data were presented as cases (%), and the chi-square test was used for comparison between groups. For measurement data, if they conformed to the normal distribution, they were described as (*x–* ± s), and the t-test was used for comparison between groups. For multivariable logistic regression: Variables with *p* < 0.10 in univariate analysis were selected as candidate covariates, including age, sex, smoking index, duration of disease, hypertension, coronary atherosclerotic heart disease, diabetes mellitus, cerebral infarction, plasma Kyn, Trp, KTR, and Arg. These covariates were entered into the multivariable logistic regression model to identify independent influencing factors for 1-year poor prognosis. For data with skewed distribution, they were described as [M (Q1, Q3)], and the non - parametric Kruskal-Wallis test was used for comparison between groups. Spearman correlation analysis was used to analyze the correlation between KTR and Arg levels and pulmonary function. Receiver operating characteristic (ROC) curves were drawn to evaluate the predictive efficacy of research indicators. A result with *p* < 0.05 was considered statistically significant.

## 3 Results

### 3.1 General clinical characteristics

Compared with the group with good prognosis, the group with poor prognosis had a higher proportion of GOLD grade 3/4. There were no statistically significant differences between the two groups in general information such as age, gender, and smoking exposure. See Table 1 for details.

### 3.2 Differences in plasma Kyn, Trp, KTR, and Arg levels among AECOPD patients with different prognoses and healthy controls

**Figure 2:**
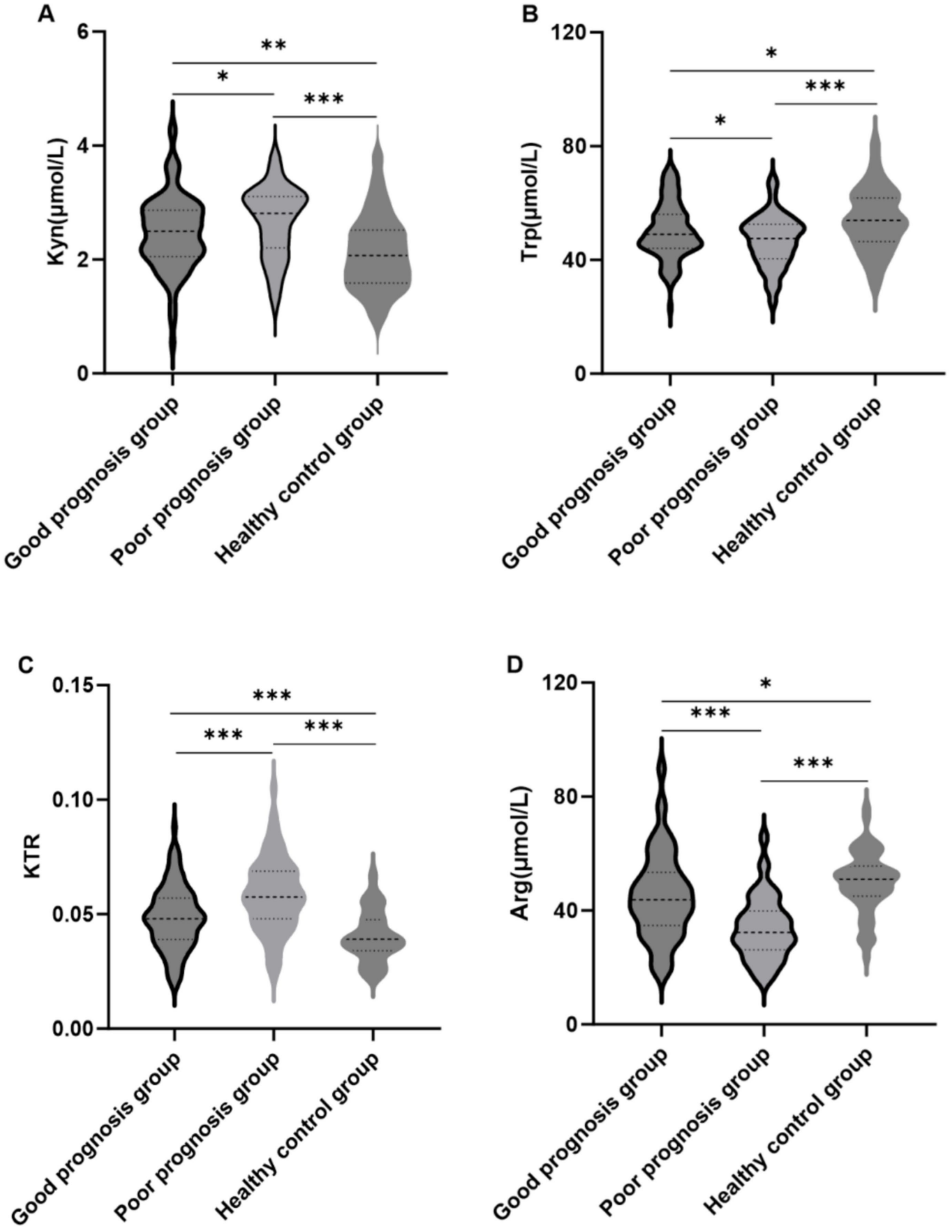
Comparison of plasma Kyn, Trp, KTR, and Arg levels among the good prognosis, poor prognosis, and healthy control groups. **(A)** Kyn, **(B)** Trp, **(C)** KTR, **(D)** Arg. ^*^p < 0.05, ^**^p < 0.01, ^***^p < 0.001.

Violin plots compared plasma Kyn, Trp, KTR, and Arg levels among good prognosis, poor prognosis, and healthy control groups (Figure 2). Compared with healthy controls, both AECOPD groups showed elevated Kyn and KTR, reduced Trp and Arg (all *p* < 0.05). The poor prognosis group exhibited higher KTR and lower Arg and Trp than the good prognosis group (all *p* < 0.05), indicating more severe metabolic dysregulation (Figures 2A–D).

### 3.3 Correlation between plasma KTR and Arg levels and pulmonary function grades

The results of Spearman correlation analysis showed that the GOLD grade was positively correlated with the plasma KTR level (*r* = 0.747, *p* < 0.001; Table 2) and negatively correlated with the Arg level (*r* = −0.638, *p* < 0.001; Table 2).

**Table 2:** Correlation between plasma KTR and Arg levels and GOLD classification.

Variable	GOLD classification	
	*r* value	*p* value
KTR	0.747	<0.001
Arg	−0.638	<0.001

KTR, Kynurenine to tryptophan ratio; Arg, Arginine; GOLD, Global Initiative for Chronic Obstructive Lung Disease.

### 3.4 Correlation between plasma KTR and Arg levels in AECOPD patients, stratified by prognosis

**Figure 3:**
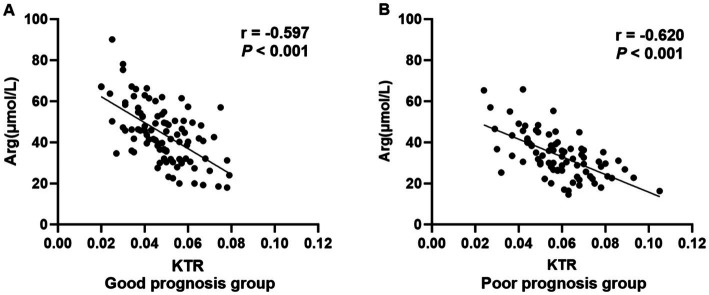
Correlation between plasma KTR and Arg levels, stratified by prognosis in patients with AECOPD **(A,B)**.

Stratified scatter plots with regression lines were constructed to visualize the correlation between KTR and Arg levels in AECOPD patients, grouped by prognosis (Figure 3). In the good prognosis group, a significant negative correlation was observed (*r* = −0.597, *p* < 0.001). Similarly, the poor prognosis group also showed a strong negative correlation (*r* = −0.620, *p* < 0.001), indicating a consistent inverse metabolic relationship between KTR and Arg across both prognostic subgroups.

### 3.5 Analysis of influencing factors for poor prognosis within 1 year in patients with AECOPD

The research indicators and clinical covariates (age, sex, smoking index, course of disease, hypertension, coronary atherosclerotic heart disease, diabetes mellitus, cerebral infarction, GOLD classification) were included in the Logistic regression analysis and adjusted for multiple factors. The results showed that the increased levels of Kyn and KTR were risk factors for poor prognosis of patients with AECOPD within 1 year after discharge, while the increased levels of Trp and Arg were protective factors (*p* < 0.05, Table 3).

**Table 3:** Multivariate logistic regression analysis of poor prognosis in patients with AECOPD.

Variable	*β*	*SE*	*Wald χ^2^*	*p* value	OR	95% CI
Age (year)	0.023	0.019	1.485	0.223	1.024	0.986–1.063
Sex (1 = male, 0 = female)	0.663	0.365	3.296	0.069	0.515	0.252–1.054
Duration of disease (year)	0.007	0.016	0.190	0.663	1.007	0.977–1.038
Hypertension (1 = Yes, 0 = No)	0.474	0.346	1.882	0.170	1.607	0.816–3.162
Coronary atherosclerotic heart disease (1 = Yes,0 = No)	0.736	0.472	2.429	0.119	0.479	0.190–1.209
Diabetes mellitus (1 = Yes, 0 = No)	0.988	0.599	2.714	0.099	2.685	0.829–8.692
Cerebral infarction (1 = Yes, 0 = No)	0.773	0.429	3.248	0.072	2.166	0.935–5.021
Smoking index (pack-years)	0.001	0.001	3.275	0.070	1.001	1.000–1.002
GOLD classification (1 = Grade 1/2, 2 = Grade 3/4)	0.731	0.312	5.497	0.019	2.078	1.127–3.830
Kyn (μmol/L)	0.565	0.248	5.169	0.023	1.759	1.081–2.862
Trp (μmol/L)	0.800	0.371	4.654	0.031	0.449	0.217–0.929
KTR (Kyn/Trp ratio, dimensionless)	1.052	0.368	8.518	0.004	2.862	1.391–5.890
Arg (μmol/L)	0.171	0.028	36.135	<0.001	0.843	0.797–0.891

Covariates included in the model: age, sex, course of disease, hypertension, coronary atherosclerotic heart disease, diabetes mellitus, cerebral infarction, smoking index, GOLD classification, Kyn, Trp, KTR, and Arg. Variable selection: variables with *p* < 0.10 in univariate analysis were entered into the model. KTR, Kynurenine to tryptophan ratio; Kyn, Kynurenine, Trp, Tryptophan, Arg, Arginine.

### 3.6 Comparison of diagnostic efficacy of KTR and Arg in predicting poor prognosis of AECOPD

**Figure 4:**
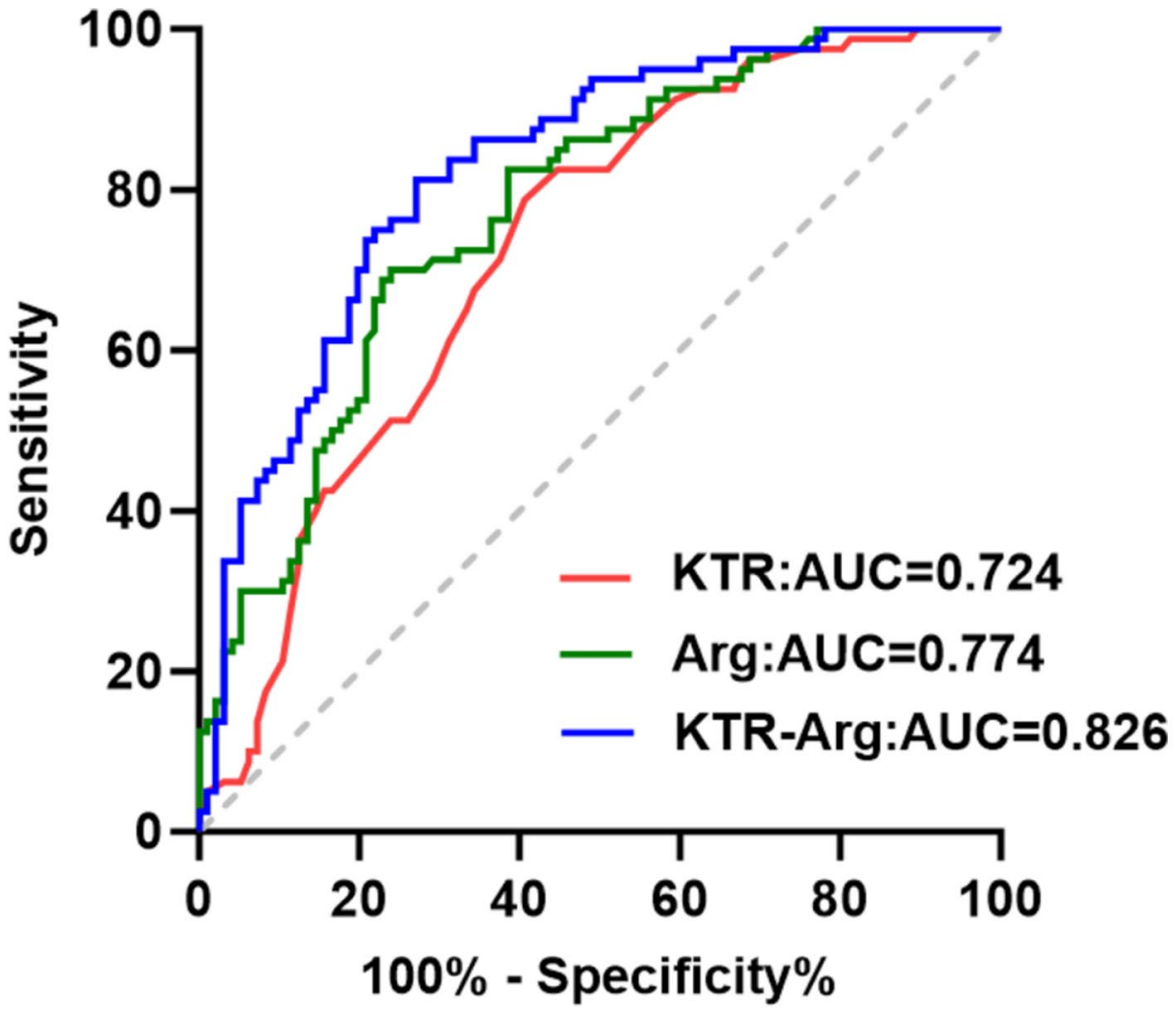
ROC curves of KTR and Arg alone and in combination for predicting poor prognosis in patients with AECOPD.

The AUC of KTR in predicting the poor prognosis of AECOPD was 0.724 (sensitivity: 78.70%, specificity: 59.40%; Figure 4; Table 4); the AUC of Arg was 0.774 (sensitivity: 70.00%, specificity: 76.00%, Figure 4; Table 4). When the two were combined for prediction, the AUC reached the maximum value of 0.826, with a sensitivity of 81.25% and the highest positive likelihood ratio of 3.00 (Figure 4; Table 4), which was significantly better than the predictive value of a single indicator.

**Table 4:** Comparison of diagnostic efficacy of KTR and Arg in predicting poor prognosis of AECOPD.

Variable	Sensitivity (%)	Specificity (%)	PLR	NLR	AUC (95% CI)	Cut-off value
KTR	78.70	59.40	1.94	0.36	0.724 (0.652–0.789)	0.05
Arg	70.00	76.00	2.92	0.39	0.774 (0.705–0.834)	34.27 μmol/L
KTR + Arg	81.25	72.92	3.00	0.26	0.826 (0.762–0.879)	-

KTR, Kynurenine to tryptophan ratio; Kyn, Kynurenine, Arg, Arginine; PLR, Positive likelihood ratio; NLR, Negative likelihood ratio.

## 4 Discussion

Frequent acute exacerbations of COPD can lead to continuous decline in patients’ pulmonary function and immune function, resulting in extremely poor prognosis. As the basic components of immune proteins, amino acids are closely related to the activation and proliferation of granulocytes, lymphocytes, and NK cells in the body under inflammatory conditions (11). Therefore, evaluating the disease outcome of patients with AECOPD using changes in amino acid metabolite levels is of great significance for guiding clinical treatment and predicting prognosis.

AECOPD patients with poor prognosis had a markedly higher proportion of GOLD grade 3/4 cases than those with good prognosis. Corresponding to this severe pulmonary function phenotype, violin plot analysis revealed distinct tryptophan and arginine metabolic perturbations in AECOPD patients relative to healthy controls, with elevated Kyn and KTR and reduced Trp and Arg (all *p* < 0.05). Notably, these metabolic dysregulations were further exacerbated in the poor prognosis cohort, which had a higher KTR and more pronounced reductions in Trp and Arg (all *p* < 0.05), reflecting more severe disturbances in these metabolic pathways in patients with unfavorable clinical outcomes and impaired pulmonary function. This close link between metabolic dysregulation and poor clinical outcomes was further supported by our 1-year follow-up data for AECOPD patients. The follow-up results showed that the incidence of poor prognosis was 45.45% (74 cases were hospitalized again, and 6 cases died because of treatment failure.), which was similar to the results of previous studies (12, 13). Patients with AECOPD are in a state of strong inflammatory response, which leads to an increase in the level of indoleamine 2,3-dioxygenase (IDO) in the blood. This accelerates the conversion of Trp to Kyn, resulting in Trp depletion and elevated KTR. Since Trp is an essential amino acid for T cell proliferation and differentiation, a decrease in Trp levels impairs airway anti-inflammatory capacity, triggering an inflammatory cascade amplification effect and accelerating the decline of pulmonary function.

Arg can reduce the adhesion between leukocytes and endothelial cells, thereby decreasing leukocyte extravasation, which exerts a protective effect against local organ damage caused by inflammation (14). A study by Mohammadi Z et al. (15) demonstrated that Arg can significantly inhibit the release of pro-inflammatory cytokines, including C-reactive protein, interleukin-6, and tumor necrosis factor-*α*, thus reducing inflammatory injury in the body. Zinellu A et al. (16) conducted a study involving 43 patients with mild and moderate COPD and concluded that the KTR could serve as a biomarker for distinguishing mild from moderate COPD. In the present study, we included AECOPD patients with pulmonary function ranging from grade 1 (mild) to grade 4 (very severe), and performed correlation analysis with KTR levels. This provides updated evidence regarding the correlation between changes in Trp metabolite levels and the severity of pulmonary function impairment-specifically, higher KTR levels are associated with more severe pulmonary function damage.

We also found that higher Arg levels correlate with better pulmonary function. A potential explanation for this is that Arg is the sole precursor of nitric oxide (NO), which plays a crucial role in regulating the tone of airway and vascular smooth muscle in the lungs. Additionally, NO can reduce neutrophil recruitment in the lungs, decrease mucus secretion and collagen synthesis, and delay the progression of pulmonary injury. Notably, a consistent strong negative correlation between plasma KTR and Arg levels was observed across both good and poor prognosis subgroups of AECOPD patients (good prognosis: *r* = −0.597, *p* < 0.001; poor prognosis: *r* = −0.620, *p* < 0.001), suggesting an inherent inverse metabolic relationship between these two indicators in the AECOPD population. This synergistic metabolic dysregulation-elevated KTR accompanied by reduced Arg-may jointly exacerbate the inflammatory response and pulmonary function impairment, further contributing to poor clinical outcomes. Collectively, these findings suggest that both KTR and Arg may serve as biomarkers for evaluating the severity of pulmonary function impairment in patients with COPD. Consistent with previous findings, higher KTR levels and lower Arg levels are associated with poor disease prognosis (17–19). In this study, multivariate logistic regression analysis revealed that elevated KTR levels are a risk factor for short-term poor prognosis in AECOPD, while increased Arg levels act as a protective factor. COPD is a chronic disease characterized by persistent airway inflammation. When Trp and Arg levels decrease or are excessively depleted during acute exacerbation, adverse events may occur. Correale J et al. (20) demonstrated that enhanced inflammation leads to elevated Kyn levels, which inhibit the immune system response, causing inactivation and apoptosis of Th1 cells and effector T cells, thereby impairing the body’s ability to eliminate pathogen infections. Ma C et al. (21) indicated that Arg can suppress the production of inflammatory mediators and alleviate pulmonary injury caused by inflammatory responses. Results from ROC curve analysis showed that the combined detection of KTR and Arg yielded the highest AUC of 0.826 for predicting 1-year poor prognosis in AECOPD patients, with a sensitivity of 81.25% and a specificity of 72.92%. This performance was superior to that of either biomarker alone, further confirming that KTR and Arg have high clinical value for predicting the prognosis of AECOPD patients.

This study has several limitations. First, as a single-center study with a relatively small sample size, it is subject to geographical and selection biases, which may reduce statistical power and limit the generalizability of the conclusions, and the predictive value of KTR and Arg needs further verification in large-sample multicenter studies. Second, notable GOLD classification imbalance existed between prognostic groups; given the strong correlation between KTR and GOLD grade, disease severity may act as a potential confounding factor for the association between the studied metabolic indicators and AECOPD prognosis. Third, no detailed dietary and nutritional data were collected, and the potential impact of dietary intake on tryptophan metabolism cannot be completely excluded despite excluding patients taking amino acid drugs or supplements. Fourth, AECOPD treatment-related factors (e.g., dosage and course of systemic corticosteroid use, bronchodilator and antibiotic regimens) were not quantified or adjusted for in the analysis; these interventions that modulate inflammatory pathways and amino acid metabolism may have confounded the observed relationships between plasma KTR/Arg levels and clinical outcomes. Fifth, the specific molecular mechanisms underlying KTR and Arg’s effects on AECOPD prognosis remain unelucidated and require further basic experimental research. Finally, only baseline plasma KTR and Arg levels at admission were detected without dynamic monitoring during treatment and follow-up, so their dynamic changes in response to clinical interventions and across disease stages remain unclear, and serial measurements are needed in future studies.

## 5 Conclusion

This study demonstrates that plasma KTR and Arg levels are strongly associated with the severity of pulmonary function impairment (per GOLD classification) and clinical prognosis in patients with AECOPD, indicating their potential value as biomarkers for prognostic stratification in this patient population. The observed associations between elevated KTR, reduced Arg at admission and poor clinical outcomes highlight a meaningful metabolic link to AECOPD prognosis, which may provide a reference for further exploring prognostic assessment strategies for AECOPD patients.

## Data Availability

The original contributions presented in the study are included in the article/supplementary material, further inquiries can be directed to the corresponding author.
